# Benchmarking the high conductive two-dimensional layered structured NbS_2_, ZrS_2_, ReS_2_ and NbSe_2_ materials with zero energy bandgap (*E*_g_) for photocatalytic application: a DFT study

**DOI:** 10.1098/rsos.241560

**Published:** 2025-03-05

**Authors:** Muhammad Hasnain Jameel, Aqeela Yasin, Alaa Nihad Tuama, Abdullah Hasan Jabbar, Samreen Kousar, Mohd Zul Hilmi Mayzan, Muhammad Sufi Roslan, Arman Nawaz, Khaled Althubeiti, Mohammed Aljohani

**Affiliations:** ^1^ Department of Physics and Chemistry, Faculty of Applied Sciences and Technology (FAST), Universiti Tun Hussein Onn Malaysia, Muar, Johor 84600, Malaysia; ^2^ Ceramic and Amorphous Group (CerAm), Faculty of Applied Sciences and Technology, Pagoh Higher Education Hub, Universiti Tun Hussein Onn Malaysia, Panchor, Johor 84600, Malaysia; ^3^ School of Materials Science and Engineering and Henan Key Laboratory of Advanced Magnesium Alloy and Key Laboratory of Materials Processing and Mold Technology (Ministry of Education), Zhengzhou University, Zhengzhou, People’s Republic of China; ^4^ Department of Physics, College of Education for Pure Sciences, University of Babylon, Babylon, Iraq; ^5^ Scientific Research Center, Al-Ayen Iraqi University, Thi-Qar 64001, Iraq; ^6^ Department of Chemistry, University of Sahiwal, Sahiwal, Pakistan; ^7^ Department of Physics, University of Agriculture Faisalabad, Faisalabad 38040, Pakistan; ^8^ Department of Chemistry, College of Science, Taif University, PO Box 110, Taif 21944, Saudi Arabia

**Keywords:** two-dimensional materials, zero energy bandgap, transition metal dichalcogenides, photocatalytic applications

## Abstract

Two-dimensional materials are among the most scientifically accessible materials in material science at the beginning of the twenty-first century. There has been interest in the monolayer transition metal dichalcogenide (TMDC) family because of its large active site surface area for UV photons of light for wastewater treatment. In the present work, density functional theory (DFT) is utilized to model the optical, structural and electrical properties of TMDCs such as NbS_2_, ZrS_2_, ReS_2_ and NbSe_2_ using the GGA-PBE simulation approximation. Based on DFT calculations, it is determined that NbS_2_, ZrS_2_, ReS_2_ and NbSe_2_ have zero energy bandgap (*E*_g_). The additional gamma-active states that are generated in NbS_2_, ZrS_2_, ReS_2_ and NbSe_2_ materials aid in the construction of the conduction and valence bands, resulting in a zero *E*_g_. In the ultraviolet (UV) spectrum, the increase in optical conductance peaks from 4.5 to 15.7 suggests that the material exhibits stronger absorption or interaction with UV light due to the excitation of electronic transitions or inter-band transitions. The highest optical conductivity and absorbance of two-dimensional TMDCs NbS_2_, ZrS_2_, NbSe_2_ and ReS_2_ show 2.4 × 10^5^, 2.5 × 10^5^, 2.8 × 10^5^ and 7 × 10^5^ $\Omega^{-1}\;cm^{-1}$ , respectively. The TMDC family, including two-dimensional TMDCs NbS_2_, ZrS_2_, NbSe_2_ and ReS_2_, is known for its unique electronic and optical properties. Their layered structure and high surface area make them excellent candidates for applications involving light absorption and photodetection. These materials reduce photon recombination and improve charge transport, making them suitable for photocatalytic and photoanode applications.

## 1. Introduction

Two-dimensional materials are among the most strategically accessible materials in material science at the beginning of the twenty-first century. There has been interest in the transition metal dichalcogenide (TMDC) family because of their physical, chemical and optical characteristics [1,2]. Their high extinction coefficient, dimension, shape and tuning capability make them extremely important in a wide range of industries, including photocatalytic, digital electronics and solar energy applications [3]. The chemical composition of two-dimensional materials, the oxidation-friendly valence shell of ‘S’ with _−_ 2, and the C–S bonding atoms all contribute to their improved stability [4]. Due to their intriguing and new features, two-dimensional materials have garnered a lot of attention with a large active site surface area for the absorption of photons of light for photocatalytic application [5–7].

These materials can come across as useful in field-effect transistors, electro-catalysts, optoelectronic devices, topological insulators and other devices [8,9]. Over a couple of decades, bulk TMDCs have been extensively explored due to the possibility of synthesizing compounds with different electronic structures. At first, the MX family of non-centrosymmetric WC-type hexagonal structural compounds (MX = TaN, ZrTe, WC, MoP, TaS, TiS, TiSe, TiTe, ZrS, ZrSe, HfS, HfSe and HfTe) has attracted increased attention because of the remarkable invention [10]. Due to very resilient TiS, ZrSe and HfTe are locked by the threefold rotational symmetry of the hexagonal lattices. They precisely exist in the optical–acoustic gaps and do not overlap with other phonon bands [11].

MX_2_ compounds have layered materials in bulk form, often known as van der Waals solids. The standard notation for two-dimensional TMDCs is MX_2_, where X stands for the chalcogen (S, Te and Se) and M for a transition metal (Mo, V, Ti, Nb, Hf, W and Ta) [2]. They have poor interlayer bonding as well as powerful intralayer bonding [12–14]. Each single layer of the TMDCs is made up of three atomic layers, with two chalcogens sandwiching the transition metal. Moreover, chalcogen atoms are saturated, and they do not react very strongly. These characteristics enable the TMDCs to be achieved on individual layers, creating appropriate active edges for high conductivity [15,16]. There are no interactions in the *z*-direction, and charge carriers are confined in two dimensions (the *x*- and *y*-directions), which causes drastic changes in the characteristics of TMDC monolayers when they are isolated for photocatalytic and photoanode solar cell applications [17]. The bandgap of MoS_2_, a direct gap semiconductor, lies in the visible spectrum at about 1.9 eV [18]. The thermal conductivity of ZrTe_2_ (*E*_g_ = 109.3 cm^−1^) varies with a decrease in temperature while the material remains in a stable phase at lower temperatures. A sample was found to remain stable under laser etching up to a power of 10 mW [19]. For photocatalytic and solar cell applications, these two-dimensional WS_2_, PtS_2_, MoS_2_, WSe_2_, PtSe_2_ and MoSe_2_ semiconductor materials are suitable. The energy bandgap for WS_2_, PtS_2_ and MoS_2_ shows a decreasing trend from 1.96 to 1.507 eV, while for WSe_2_, PtSe_2_ and MoSe_2_, it is 1.34 to 0.74 eV. Compared with other materials, MoS_2_ and MoSe_2_ are preferable because of their smaller band gaps [20].

Consequently, a great deal of effort has been devoted to finding semiconducting two-dimensional TMDCs with bandgaps ≈ 0 eV to facilitate the achievement of the high on/off current ratio at room temperature [21]. The TMDCs comprise one class of two-dimensional TMDCs that satisfy this requirement. TMDCs have the same structure as graphene (as zero *E*_g_), which comprises the same behaviour as carbon atoms arranged in a hexagonal lattice [22]. TMDCs can display a wide range of electronic properties, from metallic to superconducting, depending on how M and X are coupled [23,24]. Furthermore, *E*_g_ can be tuned by adjusting thickness, tension and stacking patterns because of their retained two-dimensional structure [25,26]. One other unique property of two-dimensional layer materials is their ability to support flexible stacked van der Waals heterostructures without lattice matching due to the weak van der Waals force between layers. With the help of all these characteristics, band structures at an atomic level can be engineered in a variety of ways, yielding a vast array of novel materials appropriate for photocatalytic applications [27,28]. There is no indication that the rapid pace of TMDC material development has slowed down. The need for uniform growth with wafer-scale size at zero bandgap level continues to be the primary obstacle inhibiting the practical deployment of TMDCs in optoelectronic and solar cell applications. Additionally, there is a need for more dependable and consistent device-processing technologies because techniques created for traditional Si and Ge materials cannot be used to manufacture TMDC devices [29–31]. Particularly, research is still ongoing to determine appropriate doping techniques that preserve the two-dimensional nature of TMDCs. Achieving high carrier mobility and good contact (zero bandgap) are also essential for TMDC device optimum efficiency and photocatalytic application. Thus, for the vast implementation of TMDC-integrated electronic systems, the growth of material and consequent device processing remains the primary obstacles [32–34].

Currently, the growth of industrial electronic companies is operating based on the promising potential of TMDCs, notwithstanding the unresolved challenges in this field. Although it is unfeasible to expect TDMCs to replace Si-based complementary metal-oxide semiconductors in all applications, TMDCs’ special qualities allow us to substitute traditional materials in several current uses while also serving as inspiration for new ones, such as versatile, wearable electronics devices [35,36].

The domains of harvesting energy and a sustainable environment find great appeal in the two-dimensional TMDC family. The most important objective of this work is to add some significant results related to the properties, such as structural, optical and electrical, of TMDCs using computational simulation. It is possible to determine the suitability and efficiency of photocatalytic application using TMDC materials with large active site surface area, decreased charge carrier recombination, and excitation for absorption of photons of light. To the authors’ knowledge, this is the first attempt to use TMDCs such as NbS_2_, ZrS_2_, ReS_2_ and NbSe_2_ with large active site surface area for photocatalytic activity with zero energy bandgap (E_g_) using density functional theory (DFT)-extracted data. Theoretical DFT study of the TMDC family is elaborated in this research, which has not been reported in the previously available literature.

## 2. Computational methodology

**Figure 1.**
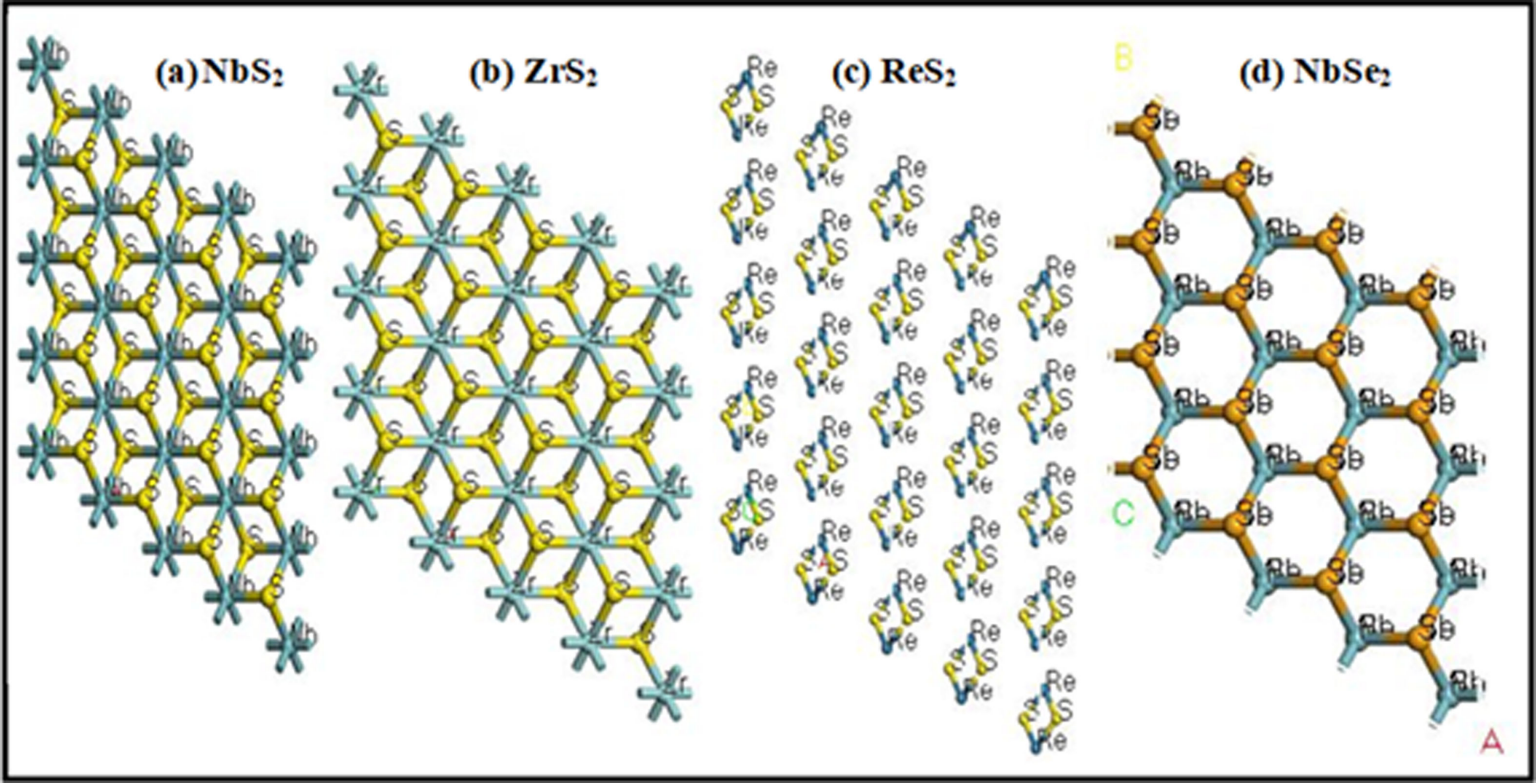
Supercell of two-dimensional layer crystal structures of (*a*) NbS_2_, (*b*) ZrS_2_, (*c*) ReS_2_ and (*d*) NbSe_2_ materials.

First-principles calculation is performed using the CASTEP simulation software [33]. The electronic structure was calculated, and the geometric structure was optimized using the generalized gradient approximation (GGA) approach. The GGA of Perdew, Burke and Ernzerhof (PBE) is widely used in DFT for calculating exchange-correlation effects [37]. The GGA functional with PBE is particularly effective for analysing crystals that contain heavy metal atoms [38]. PBE is the most versatile GGA and may be used with both solids and molecules, including metals. It is not only the best GGA for bulk solid lattice characteristics, but also the most precise for tiny organic molecules. However, being universal is crucial because, if a functional proves effective for a certain feature or system, it will unavoidably be used more broadly [39]. But, based on the random-phase approximation, the issue of CO chemisorption on transition-metal surfaces tested GGAs so far have had significant failures for either the surface energy or the adsorption energy [40]. In computational simulations using plane wave basis sets, the cut-off energy determines the maximum kinetic energy of plane waves included in the expansion of the wave function. A higher cut-off energy of 374.5 eV includes more plane waves, leading to a more accurate representation of the wave functions. The overall energy convergence of less than ${-1.02\times 10}^{3}$ eV per atom indicates that the total energy of the system has stabilized to a level below this threshold, suggesting that the structural optimization has achieved a stable minimum energy. The self-consistent convergence value of $5.76\times 10^{-4}$ eV per atom refers to the precision with which the electronic self-consistent field (SCF) calculations are converged. This means that the difference in energy between successive SCF iterations is below this value, ensuring that the electronic density and total energy are converged to a high degree of accuracy. By using USP (ultra-soft pseudo-potential) valence electron and ionic core electrostatic relations were calculated. The electrical configurations of niobium, zirconium, rhenium, sulfur and selenium are $Nb=\left[Kr\right]4d^{4}5s^{1}$ , $Zr=\left[Kr\right]4d^{2}5s^{2}$ , Re = $\left[Xe\right]4f^{14}5d^{5}6s^{2}$ , $S=\left[Ne\right]3s^{2}3p^{4}$ and $Se=\left[Ar\right]3d^{10}4s^{2}4p^{4}$ . The maximum Hellmann–Feynman force of 0.0007eV Å^−1^ indicates that the forces acting on the atoms are very small. This small force value suggests that the atomic positions are well optimized, as forces are close to zero. During the optimization, the total energy of the system is minimized with respect to the positions of the ions and the distribution of electrons. This minimization process involves adjusting the electronic structure until the forces on the atoms (Hellmann–Feynman forces) and the stress within the material meet the predefined convergence criteria, such as the maximum Hellmann–Feynman force of 0.0007eV Å^−1^ and the maximum stress of $4.19\times 10^{-2}$ . The largest possible atomic displacement is less than $1.64\times 10^{-3}$ Å. For structural optimization and electronic property calculations, the Monkhorst–Pack grid consisting of 14 × 14 × 2 k-points was utilized within Brillouin to sample. To avoid unwanted interactions in the non-periodic direction, we employed a vacuum of 35 Å along the lattice vector (here referred to as the *c* vector or *z* direction). Figure 1 presents the supercell (4 × 4 × 1) of two-dimensional TMDCs NbS_2_, ZrS_2_, ReS_2_ and NbSe_2_ materials.

## 3. Results and discussion

### 3.1. Structural study

The (two-dimensional) TMDC (NbS_2_, ZrS_2_, ReS_2_ and NbSe_2_) structured layers are simulated using the CASTEP computational program within PBE-GGA, and the atomic locations and space groups are listed in table 1. The structural study of TMDCs, such as NbS_2_, ZrS_2_, ReS_2_ and NbSe_2_, is performed using advanced computational techniques. The plane-wave pseudopotential approach is highly effective for optimizing electronic structures because it allows for accurate calculation of electronic properties, such as the density of states (DOS), band structure and charge density distribution. The GGA is used to calculate the electron exchange-correlation energy. TMDCs like NbS_2_, ZrS_2_, ReS_2_ and NbSe_2_ are layered materials that exhibit unique electronic, optical and mechanical properties, making them of significant interest in various applications, including catalysis.

**Table 1.** The space groups, lattice parameters and atomic positions of two-dimensional supercell layer structure materials.

materials	lattice parameters (Å)			atomic positions				space group
				atoms	a	b	c	
NbS_2_	*a =* 3.363	*b* = 3.363	*c* = 13.249	**Nb**	0	0	0.25	P6_3_/mmc
				**S**	0.3333	0.6667	0.3682	
ZrS_2_	*a* = 3.691	*b* = 3.691	*c* = 3.691	**Zr**	0	0	0	P3m1
				**S**	0.3333	0.6667	0.2204	
ReS_2_	*a* = 2.918	*b* = 2.918	*c* = 7.405	**Re**	0	0	0.2327	P3m1
				**S**	0.6667	0.3333	0.5475	
NbSe_2_	*a* = 3.327	*b* = 3.327	*c* = 15.609	**Nb**	0.3333	0.6667	0.25	P6_3_/mmc
				**Se**	0.3333	0.6667	0.8614	

### 3.2. Electronic properties

**Figure 2.**
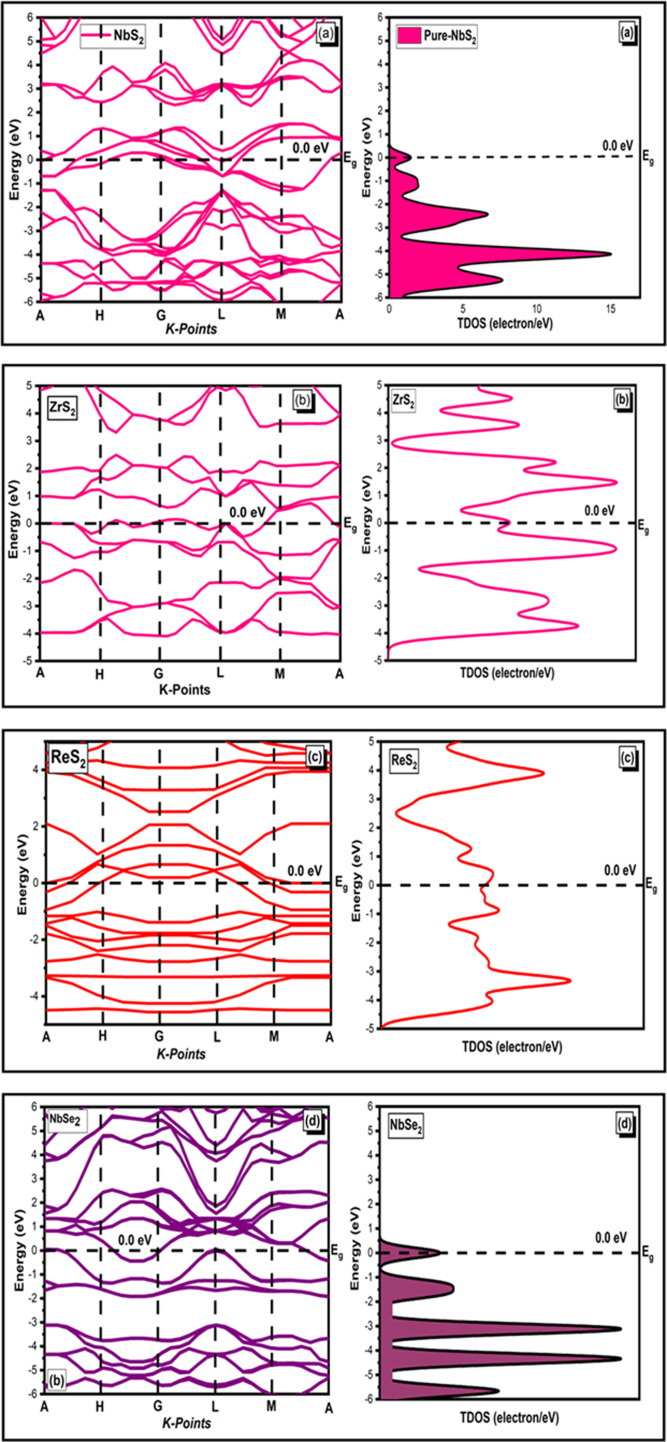
The TDOS and bandgap structures of two-dimensional (*a*) NbS_2_, (*b*) ZrS_2_, (*c*) ReS_2_ and (*d*) NbSe_2_ materials.

The DOS and partial density of states (PDOS) provide insights into the distribution of electronic states in the valence and conduction bands, which are directly linked to the material’s electrical and thermal conductivities. The energy bandgap, DOS, total density of states (TDOS) and PDOS are fundamental to understanding and predicting a material’s electronic structure and behaviour. Each of these aspects plays a crucial role in determining how a material interacts with electric fields, absorbs or emits light and conducts electricity. The electronic bandgap structure of NbS_2_, ZrS_2_, ReS_2_ and NbSe_2_ belonging to the two-dimensional TMDC family is depicted in figure 2. It has been revealed that energy bandgap structures are direct, and the conspicuous bandgap patterns of this two-dimensional TMDCs family are illustrated in figure 2. It has been determined that the NbS_2_, ZrS_2_, ReS_2_ and NbSe_2_ family exhibits an energy bandgap (*E*_g_) of 0 eV, coupled with a large active surface area, highlighting their distinctive semiconductor characteristics. The PDOS allows for the examination of ion contributions in various bandgap configurations, whereas the TDOS presents the electronic energy bandgap states per unit energy.

The newly created gamma states in the NbS_2_, ZrS_2_, ReS_2_ and NbSe_2_ family of materials are responsible for the 0 eV energy bandgap (*E*_g_) as seen by these TDOS profiles. Owing to newly created gamma states in materials S and Se, the CB is shifted along the G symmetry in the direction of the Fermi level (*E*_F_), which is a significant factor in 0 eV energy bandgap (*E*_g_) of NbS_2_, ZrS_2_, ReS_2_ and NbSe_2_ family materials. The bandgap constructions demonstrated that semiconductors with two-dimensional material had a zero bandgap. Two-dimensional crystals, such as TMDCs, provide characteristics that enhance those of graphene. As previously indicated, two-dimensional family material such as layer-structured graphene has an interesting noticeable zero bandgap (*E*_g_) value that can expand their potential in different applications such as in electronics, photocatalytic photonics and related fields [41]. One of the most significant uses of semiconductors, for instance, is in transistors for digital electronics, which are still stimulated by Moore’s Law and downsizing. Given their pristine interfaces, which are free of dangling bonds and where transport and scattering are confined to the plane of the material.

Two-dimensional materials hold significant potential to replace conventional semiconductors in ultra-scaled thin-body transistors. Their unique properties, such as atomic-scale thickness, high surface-to-volume ratio and tunable electronic characteristics, make them ideal candidates for next-generation transistor technology. Interestingly, the unique bandgap value of the NbS_2_, ZrS_2_, ReS_2_ and NbSe_2_ family of materials has demonstrated that they are an excellent candidate for solar cell and photocatalytic applications.

Defects that frequently arise as a result of the synthesis process have a significant impact on the electrical characteristics of two-dimensional materials. Point flaws, for example, result in increased photoluminescence intensity and new photoemission peaks. Localized excitons and the trapping capacity of free charge carriers are responsible for these effects. Electronic structure simulations of ZrS_2_, ReS_2_, NbSe_2_ and NbS_2_ reveal the inclusion of mid-gap states that obtain zero bandgap value and serve as scattering zones.

**Figure 3.**
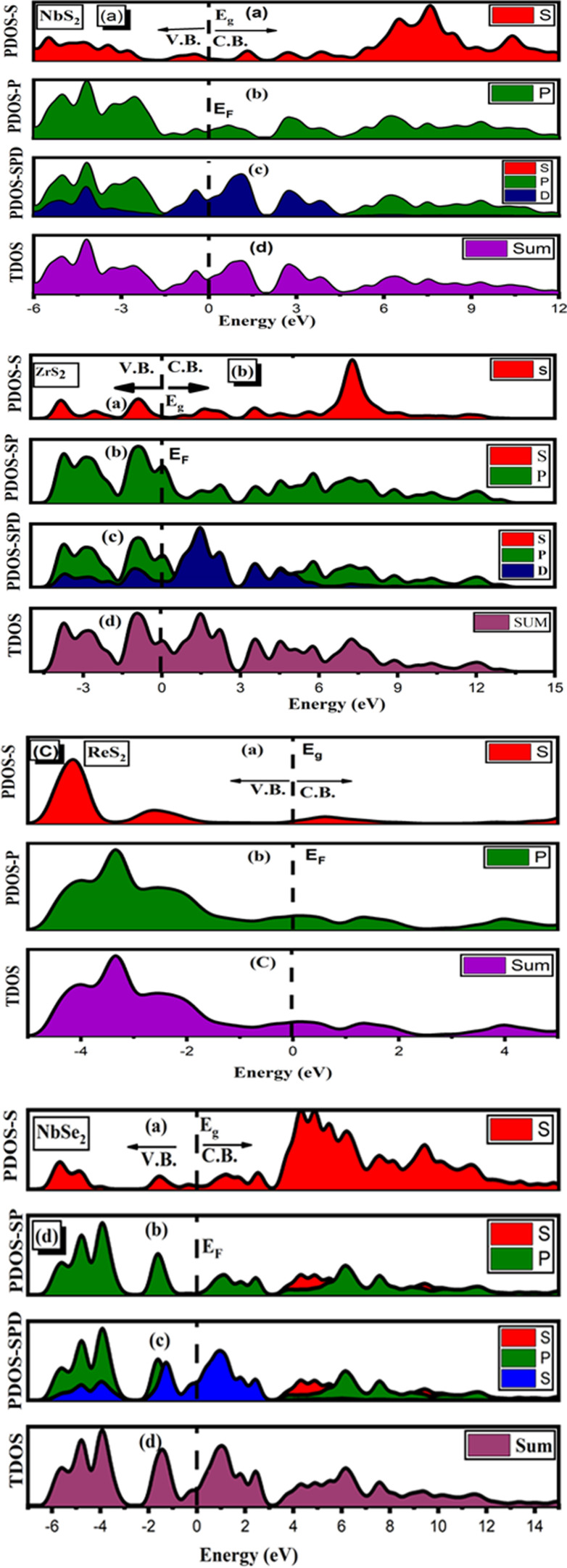
PDOS for (*a*) NbS_2_, (*b*) ZrS_2_, (*c*) ReS_2_ and (*d*) NbSe_2_ two-dimensional materials.

**Figure 4.**
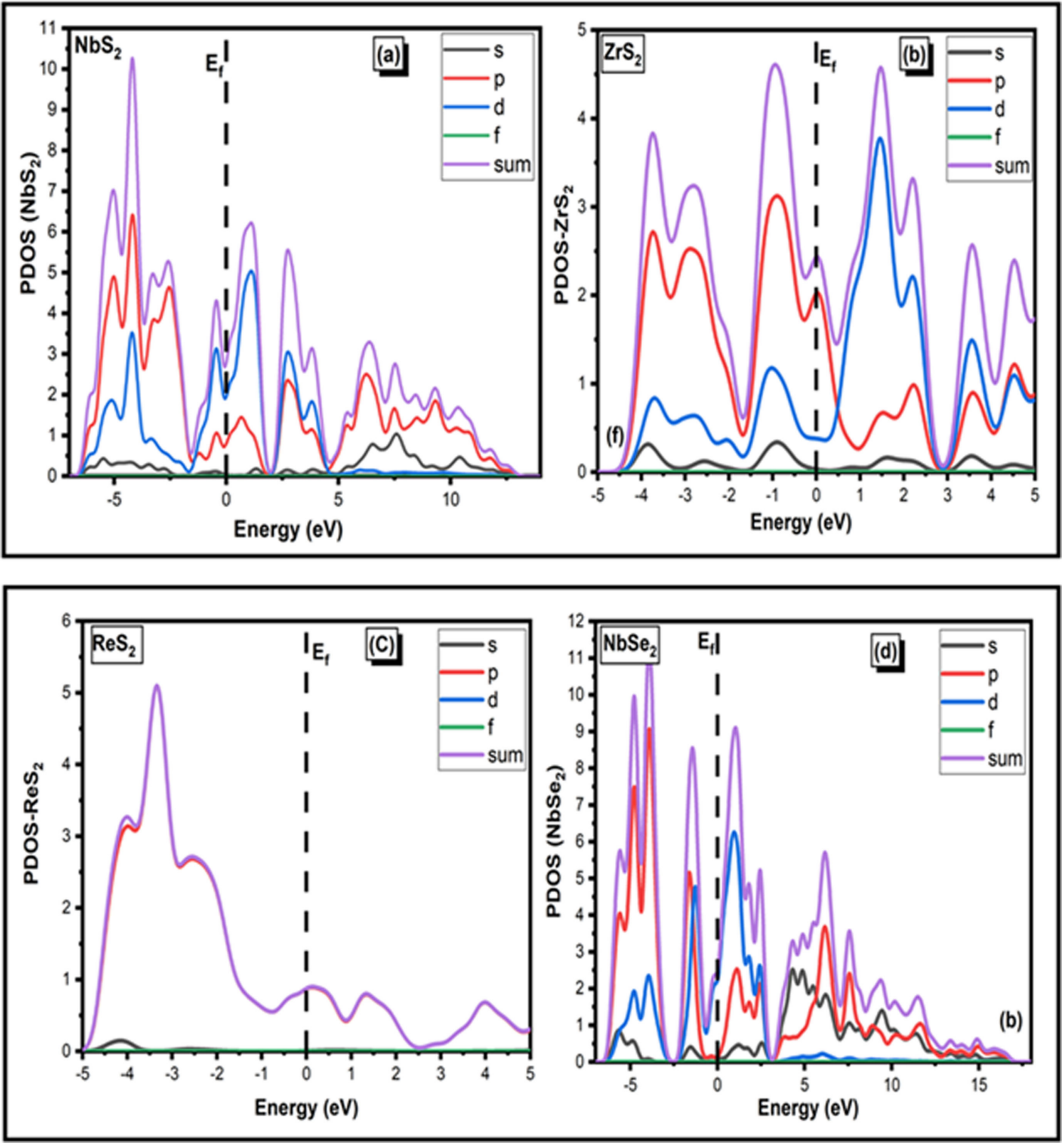
PDOS for (*a*) NbS_2_, (*b*) ZrS_2_, (*c*) ReS_2_ and (*d*) NbSe_2_ two-dimensional materials.

Element investigation of the two-dimensional TMDC family such as NbS_2_, ZrS_2_, ReS_2_ and NbSe_2_ by PDOS is displayed in figure 3. To obtain in-depth information about hybridization phenomenon as depicted in figure 2, we proceed to compute the PDOS on the Nb, Re, Se and S orbitals, as illustrated in figure 4.

Water oxidation photocatalytic activity is dependent on numerous aspects, including semiconductor photocatalyst bandgap, defect structure and crystal size [42]. Photocatalysts’ semiconducting nature is confirmed by their optical bandgap values [43]. The TMDC family of photocatalysts is an effective class that increases charge transport by reducing the energy bandgap, decreases the rate of recombination of photogenerated charge carriers, and has a large surface area for light absorption, which makes them perfect for photocatalytic and photoanode applications in cells [44]. The two-dimensional family NbS_2_, ZrS_2_, ReS_2_ and NbSe_2_ monolayers show zero bandgap semiconducting materials, according to the computed PDOSs of TDMCs.

These figures show that for all of these materials, the *p* states (shown in red) in figure 4 contribute the most in the creation of conduction band. NbSe_2_ materials have slightly more gamma states in their *p* states than in their *s*, *d* and *f* states. Therefore, in comparison to *s*, *d* and *f* states, *p* states contribute more to energy bandgap formation. NbS_2_, ZrS_2_ and NbSe_2_ show enhanced hybridization of the *s*, *p*, *d* and *f* states due to their zero-energy bandgap (*E*_g_) and extensive surface area for light absorption, as depicted in figure 3. These results suggest that these materials can be applied in photoanode and photocatalytic applications.

### 3.3. Optical properties

The optical characteristics of two-dimensional materials need to be investigated because of their significant relevance in applications involving photocatalytic processes due to large surface area and optoelectronic devices. The optical spectra of the two-dimensional TMDC family are employed to analyse how the internal structure of materials such as NbS_2_, ZrS_2_, ReS_2_ and NbSe_2_ responds to light absorption. This investigation provides insights into the materials’ interactions with light and their structural changes under optical excitation. In addition to the simultaneous interconnection of the optical conductivity process, the transition and excitation of electrons exhibit effective responses that modify optical properties. The complex dielectric function *ε*(*ω*) can be used to analyse the electric response of two-dimensional TMDCs to an electric field. The Maxwell equations and supplementary restrictions could be used to obtain the dielectric parameters *ε*(*ω*). The crystal’s optical conductivity response makes a distinction between the imaginary and real dielectric functions.

The Ehrenreich and Cohen equations are instrumental in analysing a material’s optical conductivity response as a function of photon E=ℏω energy [45]. The Ehrenreich and Cohen equations are derived from the theory of optical conductivity in solids, particularly for analysing the response of materials to electromagnetic radiation. The excited and transitional occupied and unoccupied states generate the analysed absorption spectra [46]. For the family NbS_2_, ZrS_2_, ReS_2_ and NbSe_2_, the calculated optical conductivity $\varepsilon_{1}\left(\omega \right)$ and $i\varepsilon_{2}\left(\omega \right)$ , refractive index *n*(*ω*), absorption coefficient $a\left(\omega \right)$ , tangent loss reflectivity and optical conductivity are discussed in this section. The optical properties of the family NbS_2_, ZrS_2_, ReS_2_ and NbSe_2_ materials of the dielectric mathematical formula can be analysed using the following mathematical equations [47]:


(3.1) $$\varepsilon \left(\omega \right)=\left(\varepsilon_{1}\omega \right)+і\varepsilon_{2}\left(\omega \right)$$

with real and imaginary mathematical forms $\left[\varepsilon_{2}\left(\omega \right)\right]$ ; additionally, ${[\varepsilon }_{1}\left(\omega \right)]$ . Accordingly, the following mathematical equations (3.2) and (3.3) can be used to obtain the hexagonal layer structure form of the two-dimensional family NbS_2_, ZrS_2_, ReS_2_ and NbSe_2_ materials [48]:


(3.2) $$\varepsilon_{2}\left(\omega \right)=-\frac{Ve^{2}}{2\pi \hbar m^{2}\omega^{2}}\int d^{3}k\sum_{nn^{\prime }}1<kn\left|P\right|kñ\;\;\;I^{2}f\left(k\right)\times \left(1-f\left(kñ\right)\delta \left(E_{kn}-E_{kñ\;}-\hbar \omega \right)\right).$$

The expression $\varepsilon_{2}\left(\omega \right)$ describes how the imaginary part of the dielectric function is calculated from the electronic band structure and transition matrix elements. It incorporates photon energy, electronic state occupation and the momentum matrix elements, providing a detailed picture of how a material absorbs electromagnetic radiation [49,50]:


(3.3) $$\left(\varepsilon_{1}\omega \right)=1+\frac{2}{\pi }P\int_{0}^{\infty }\frac{\omega^{\prime }\varepsilon_{2}\left(\omega^{\prime }\right)d\omega^{\prime }}{\omega^{\prime 2}-\omega^{\prime }}=n^{2}\left(\omega \right)-k^{2}\left(\omega \right).$$

**Figure 5.**
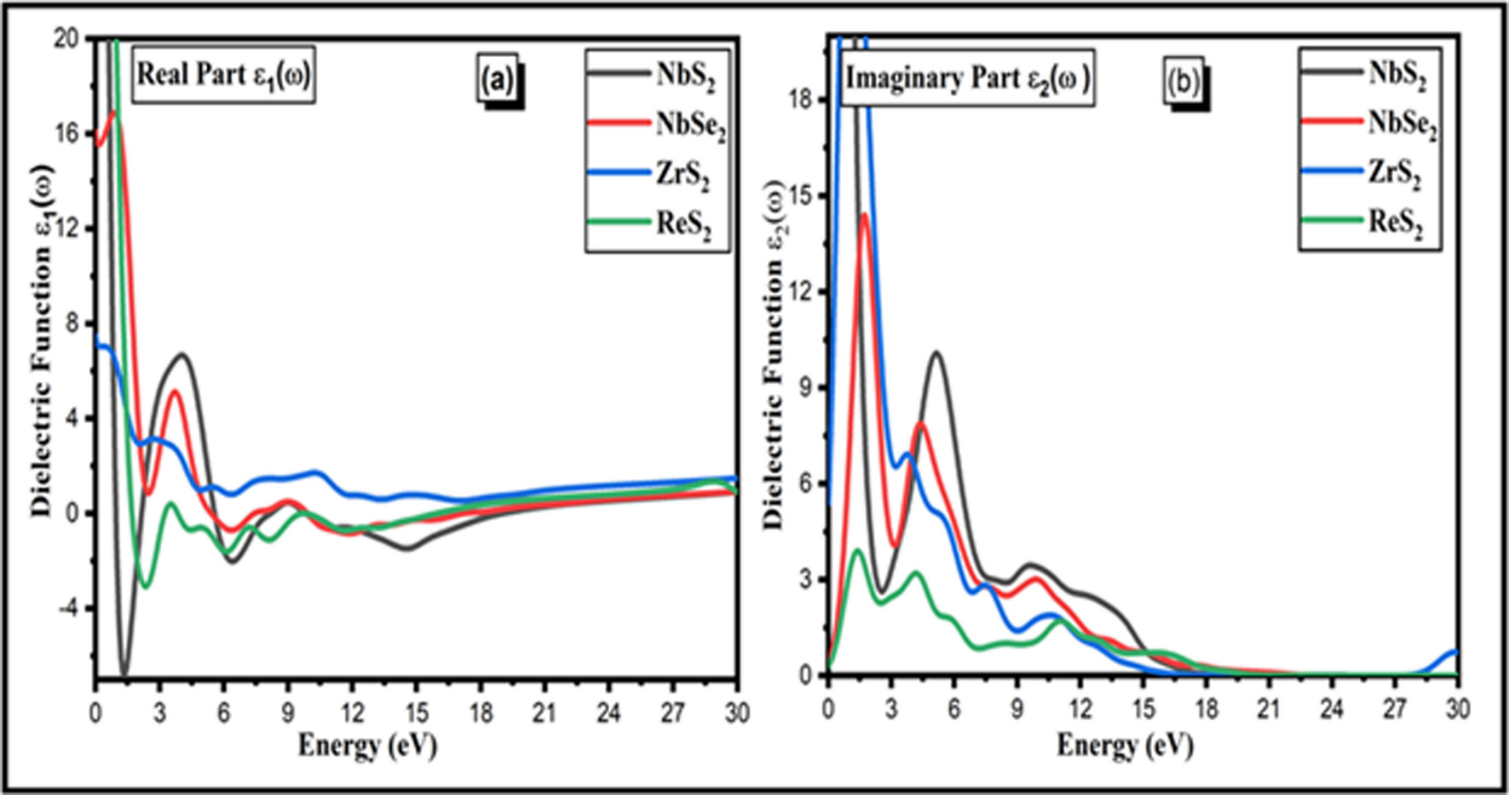
The (*a*) real and (*b*) imaginary terms of the dielectric functions of the NbS_2_, ZrS_2_, ReS_2_ and NbSe_2_ two-dimensional materials.

The intra- and inter-transition energy bands are related by dielectric functions. In the TMDC family, the real part $\left[\varepsilon_{1}\left(\omega \right)\right]$ is displayed, and the imaginary part denotes energy dissipation (loss). The principal peak of $\varepsilon_{1}\left(\omega \right)$ for two-dimensional layer structures of NbS_2_ indicates that the maximum reaches around 7 at 4 eV while the principal peak of $\varepsilon_{1}\left(\omega \right)$ for NbSe_2_ indicates that the maximum approaches around 5 at 4 eV as shown in figure 5. The major peak of $\varepsilon_{1}\left(\omega \right)$ for ZrS_2_ indicates that the maximum reaches around 3.9 at 4 eV while the main peaks of $\varepsilon_{1}\left(\omega \right)$ for ReS_2_ indicates that the maximum approaches around 0.6 at 4 eV. The ZrS_2_, ReS_2_, NbSe_2_ and NbS_2_ curves all show the same pattern. The complex dielectric function $\varepsilon_{1}\left(\omega \right)$ is depicted in figure 5*a*. This function is generated by the imaginary function i $\varepsilon_{2}\left(\omega \right)$ of the dielectric factor using the mathematical formulation of Kramer and Kronig [51]. The main peaks for the two-dimensional layer structures NbS_2_ are 10 at 4 eV, NbSe_2_ are 8, ZrS_2_ are 7 at r eV and ReS_2_ are 3.5 at 4 eV. The two-dimensional layer structure of NbS_2_ shows large peak 10 at 4 eV as compared with other materials. According to findings, these layered structured materials can be used in photocatalytic and photoanode applications.

#### 3.3.1. Refractive index

**Figure 6.**
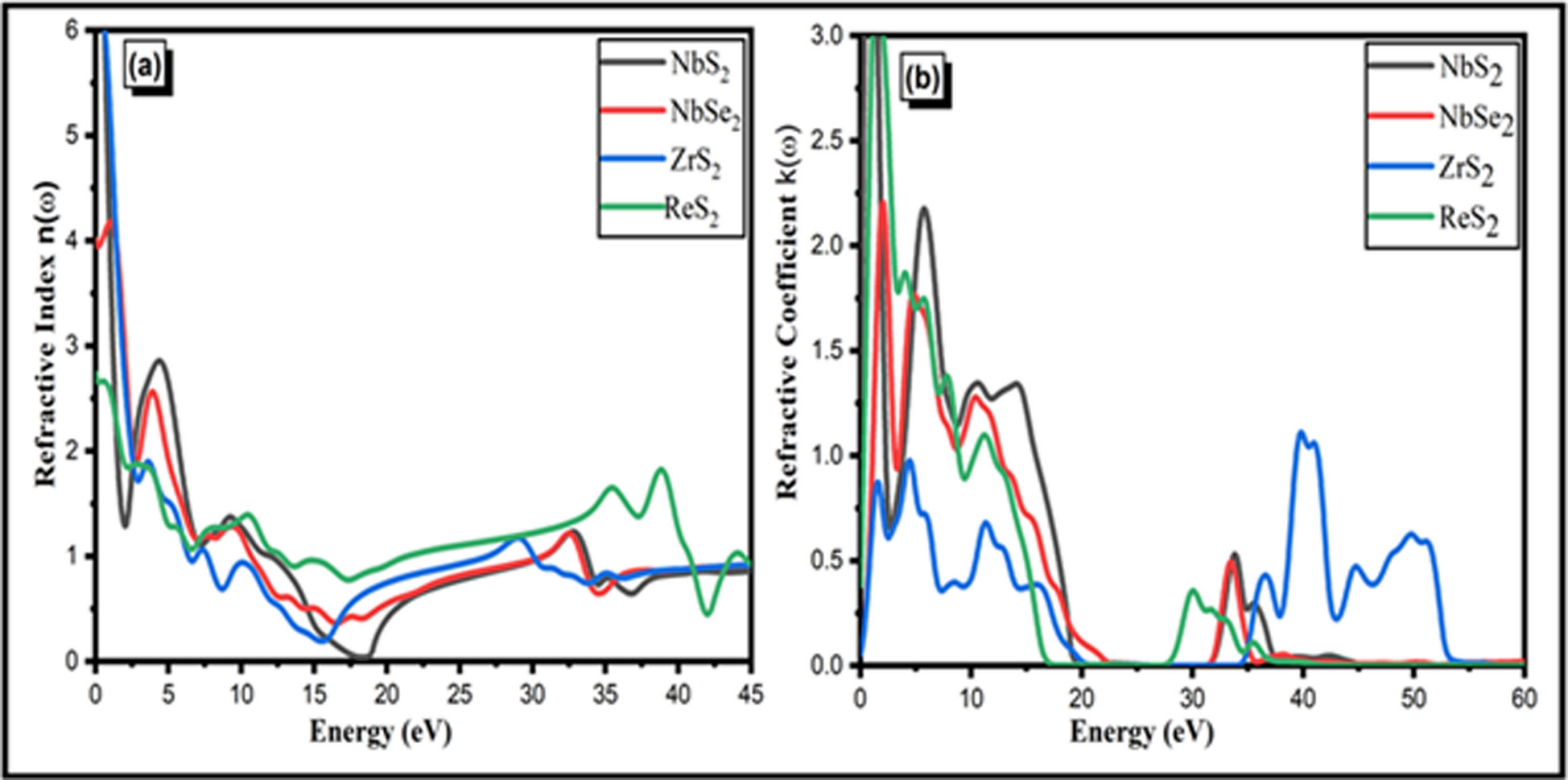
(*a*) The refractive index and (*b*) refractive coefficient of the dielectric functions of the NbS_2_, ZrS_2_, ReS_2_ and NbSe_2_ two-dimensional materials.


The electromagnetic radiation absorption and optical transparency assessment are demonstrated in figure 6 through a close examination of the refractive index *n*(*w*) and extinction coefficient *k*(*w*). Figure 5 shows the responses of the extinction coefficient *k*(*w*) and refractive index *n*(*w*) in the 0−60 eV range. The dielectric functions ${[\varepsilon }_{1}\left(\omega \right)]$ and $[\varepsilon_{2}\left(\omega \right)]$ are fundamental for understanding the optical properties of materials as they vary with frequency. They provide insights into the material’s interaction with electromagnetic waves, and they are related to the complex refractive index *n*(*ω*). They provide insight into the material’s dispersion and absorption properties and are directly related to the complex refractive index *n*(*ω*) [52–54]:

(3.4) $$\tilde{n}\left(\omega \right)=n\left(\omega \right)+іk\left(\omega \right)=\epsilon^{1/2}={\left(\varepsilon_{1}+і\varepsilon_{2}\right)}^{1/2}.$$

In this context, the optical properties of materials are often described using the extinction coefficient *k*(ω) and *n*(ω):


(3.5) $$k\left(\omega \right)=\frac{I\left(\omega \right)}{2\left(\omega \right)}.$$

The extinction coefficient k(ω) is given by the equation (ω)=I(ω)/2(ω) , where $I\left(\omega \right)$ represents the imaginary part of the dielectric function, $\left(\omega \right)$ and is the photon energy. This relationship is useful for analysing how materials absorb and scatter light at different photon energies [54,55].


(3.6) $$r\left(\omega \right)=\frac{n+iK-1}{n+iK+1}.$$

The above equation (3.6) is used to calculate the complex reflection coefficient of a material, where n is the real part of the refractive index and K is the extinction coefficient. This coefficient describes how light is reflected by the material, incorporating both the amplitude and phase changes of the reflected light.

The refractive index (*n*) of the two-dimensional NbS_2_, ZrS_2_, ReS_2_ and NbSe_2_ materials at 0 eV are 6, 4, 5.9 and 2.7, respectively. Maximum refractive index is exhibited by two-dimensional NbS_2_ and ZrS_2_ in comparison with other materials. When compared with other materials, NbS_2_ and ZrS_2_ are the most effective materials. While the refractive peaks are declining, they have sharply grown up to energy 1−17 eV. In the energy range from 0 to 60 eV, the refractive index *n*(*ω*), which corresponds to the different frequencies of the inner-transition band, varies between 0 and 6. A lower refractive index in this range indicates reduced polarization within the material for lower photon energies. As the energy increases, the refractive index increases, reflecting stronger interactions and greater polarization effects at higher energies.

#### 3.3.2. Absorbance and energy loss

The optical characteristics of TMDCs have been the subject of extensive research in recent years. Nonetheless, spectrum reflectance, differential reflectance, spectral absorbance and absorbance and differential transmittance are the main topics of consideration in most studies. The majority of these studies rely heavily on experimental research with little to no simulations conducted. The majority of studies on the optical characteristics of TMDCs lack a definitive value for extinction coefficients and refractive indices, despite extensive research on the subject. Furthermore, there is a wide range in the data acquired regarding the optical properties of TMDCs, and the majority of research involving estimations of reflectance, absorbance and refractive indices does not agree with one another.

The wavelength-based reflectance (*R*), absorbance (*A*) and transmittance (*T*) under normal incidence circumstances are provided by [55]:


(3.7) $$R=\left[{\left(n-1\right)}^{2}+K^{2}\right]/\left[{\left(n+1\right)}^{2}+K^{2}\right],$$

(3.8) $$T=\left(1-R\right)e^{-\alpha t}.$$

The absorption coefficient, denoted as *α* in equation (3.10), can be computed using the following equation [56]:


(3.9) $$\alpha =\frac{4\pi K}{\lambda }.$$

Wavelength λ represents the wavelength of the incident photon, measured in nanometres (nm). It is related to the energy of the photon by the equation E=hc/λ , where *h* is Planck’s constant and *c* is the speed of light.

In this case, λ is the wavelength (in nm) of the photon incident on the TMDC material and *K* is the extinction coefficient of the TMDCs.


(3.10) A=1−R−T.

The calculations above, which depend on the incident photon energy and material thickness, were carried out for monolayer two-dimensional NbS_2_, ZrS_2_, ReS_2_ and NbSe_2_ TMDCs.

**Figure 7.**
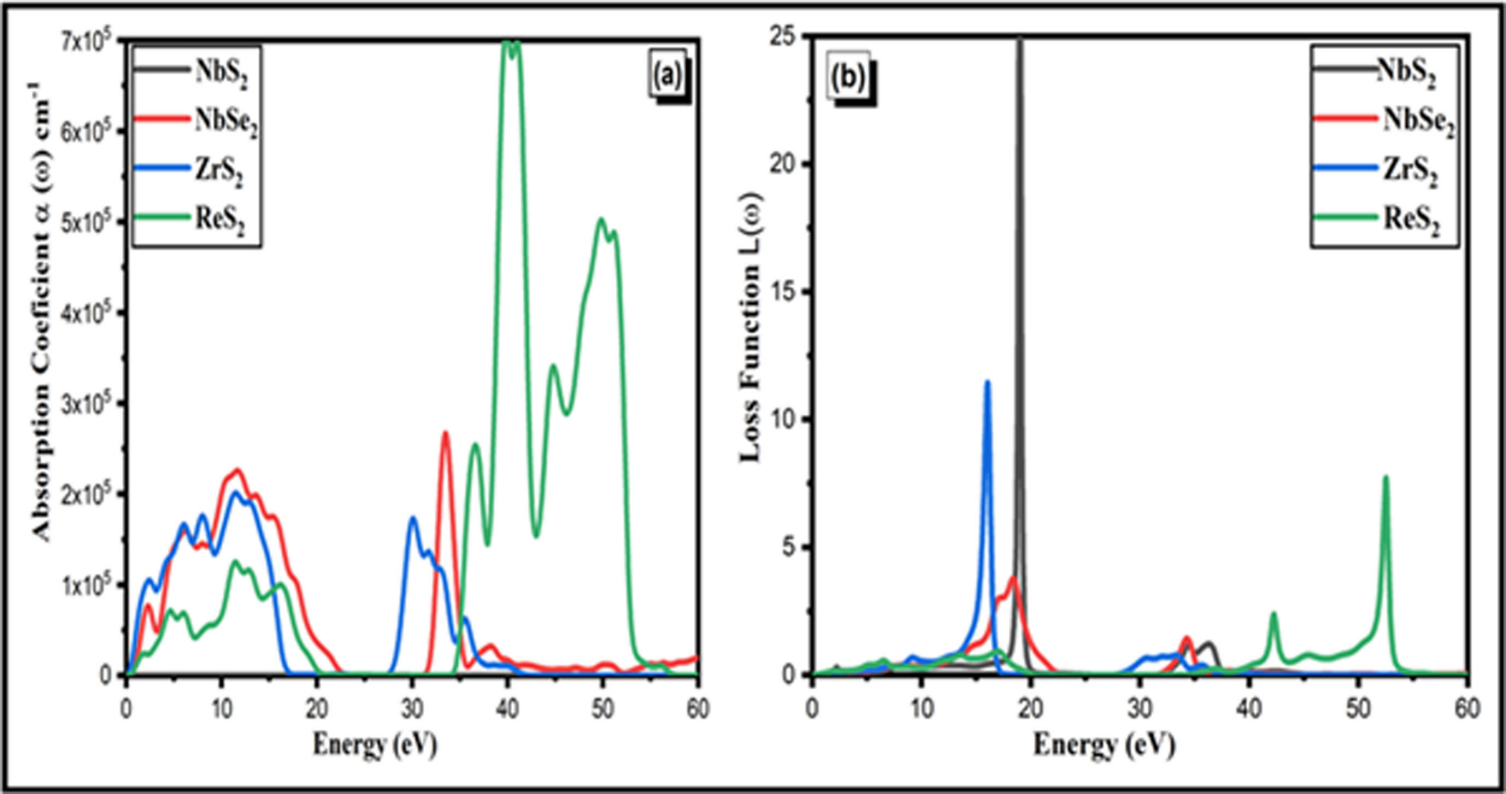
(*a*) The absorption coefficient and (*b*) energy loss function of the two-dimensional NbS_2_, ZrS_2_, ReS_2_ and NbSe_2_ materials.

Absorption in semiconductors is the process by which a material absorbs photons with energy (*E* = *ħω*) equal to or greater than its bandgap. The energy loss function *L*(ω), as shown in figure 7*b*, quantifies the energy loss per unit length of the material due to the interaction with incident photons. It is a key parameter in understanding how materials dissipate energy through processes like absorption and scattering. As seen in figure 6*a*, the absorbance of two-dimensional NbSe_2_, ZrS_2_ and ReS_2_ is rapidly increasing. All of the absorption peaks have a minor shift in the direction of higher energy values. The absorption coefficients *α*(*w*) for the TMDC materials NbSe_2_, ZrS_2_ and ReS_2_ are 2.6 × 10^5^, 2.1 × 10^5^ and 7 × 10^5^, respectively, as seen in figure 7*a*. In comparison with other materials, two-dimensional NbSe_2_ and ReS_2_ exhibit the maximum absorption at 2.6 × 10^5^ and 7 × 10^5^ cm^−1^, respectively. When it comes to absorption, these two materials are better than others. The absorption coefficient *α*(*w*) shows a shift towards higher photon energies (10–50 eV) due to the material’s small or zero bandgap. This indicates that the material absorbs light more effectively at these higher energies, reflecting its electronic structure and interaction with photons in that range. These two-dimensional NbS_2_, ZrS_2_, ReS_2_ and NbSe_2_ semiconductor materials are appropriate for photocatalytic and photoanode applications, as demonstrated by their absorption behaviour.

#### 3.3.3. Optical conductivity and reflectivity

Theoretical initiatives to quantitatively characterize two-dimensional materials in terms of their optical conductivity, a property connected to absorption, have also emerged as a result of growing interest in TMDCs. Though there is little experimental data on monolayer optical conductivities, there are a few reports on basic optical properties such as complex refractive indices, optical conductivity, reflectance and absorption coefficients. Optical conductivity is used in the photoelectric effect to demonstrate the conductance of photogenerated electrons. Electromagnetic radiation disrupts the connection between particles. Thus, in the present work, we directly evaluate the optical conductivity and other optical characteristics of monolayer two-dimensional NbS_2_, NbSe_2_, ZrS2 and ReS_2_ TMDCs utilizing tandem differential absorption and reflection measurements. Consequently, these observations have made it possible to determine each material’s absolute absorptivity as well as the related frequency-dependent refractive indices, reflectivity and optical conductivities.

**Figure 8.**
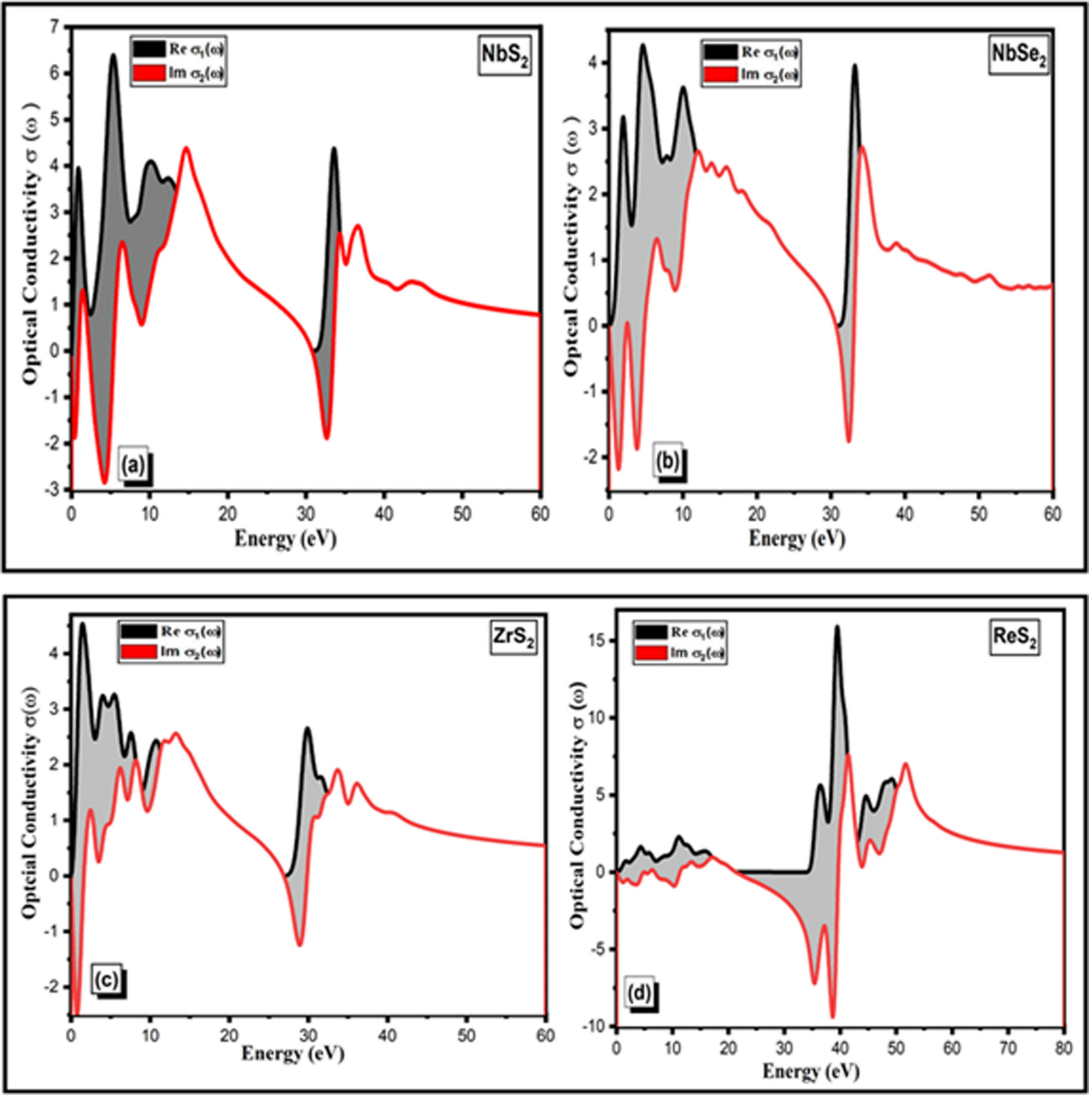
Optical conductivity of two-dimensional (*a*) NbS_2_, (*b*) NbSe_2_, (*c*) ZrS_2_ and (*d*) ReS_2_ TMDCs materials.

The optical conductivity of the two-dimensional TMDCs NbS_2_, NbSe_2_, ZrS_2_ and ReS_2_ within the 0−60 eV range varies, with maximum values of 6.5, 4.3, 4.6 and 15.5 Ω^−1^ cm^−1^, respectively, as shown in figure 8. These values indicate the materials’ varying efficiency in conducting electricity in response to electromagnetic radiation, with ReS₂ exhibiting the highest conductivity among them. The two-dimensional TMDC family’s actual conductivity response $\sigma_{1}(\omega )$ initially increases from 0 to 60 eV, but in the case of ReS_2_, it increases at 40 eV and dramatically decreases after 50 eV. Nevertheless, the highest value of 4.5, 2.7, 2.6 and 5.5 Ω^−1^cm^−1^ at 30 and 45 eV is shown by the imaginary portion of conductivity $\sigma_{2}(\omega )$ , of two-dimensional materials, respectively. Comparing two-dimensional ZrS_2_ and ReS_2_ to other NbS_2_ and NbSe_2_ materials, the maximal optical conductivity peaks are 4.6 and 15.5 Ω^−1^ cm^−1^, respectively. ZrS_2_ and NbSe_2_ are more effective than other materials. These two-dimensional NbS_2_, NbSe_2_, ZrS_2_ and ReS_2_ semiconductor materials are suitable for photocatalytic application, according to the optical conductivity results.

**Figure 9.**
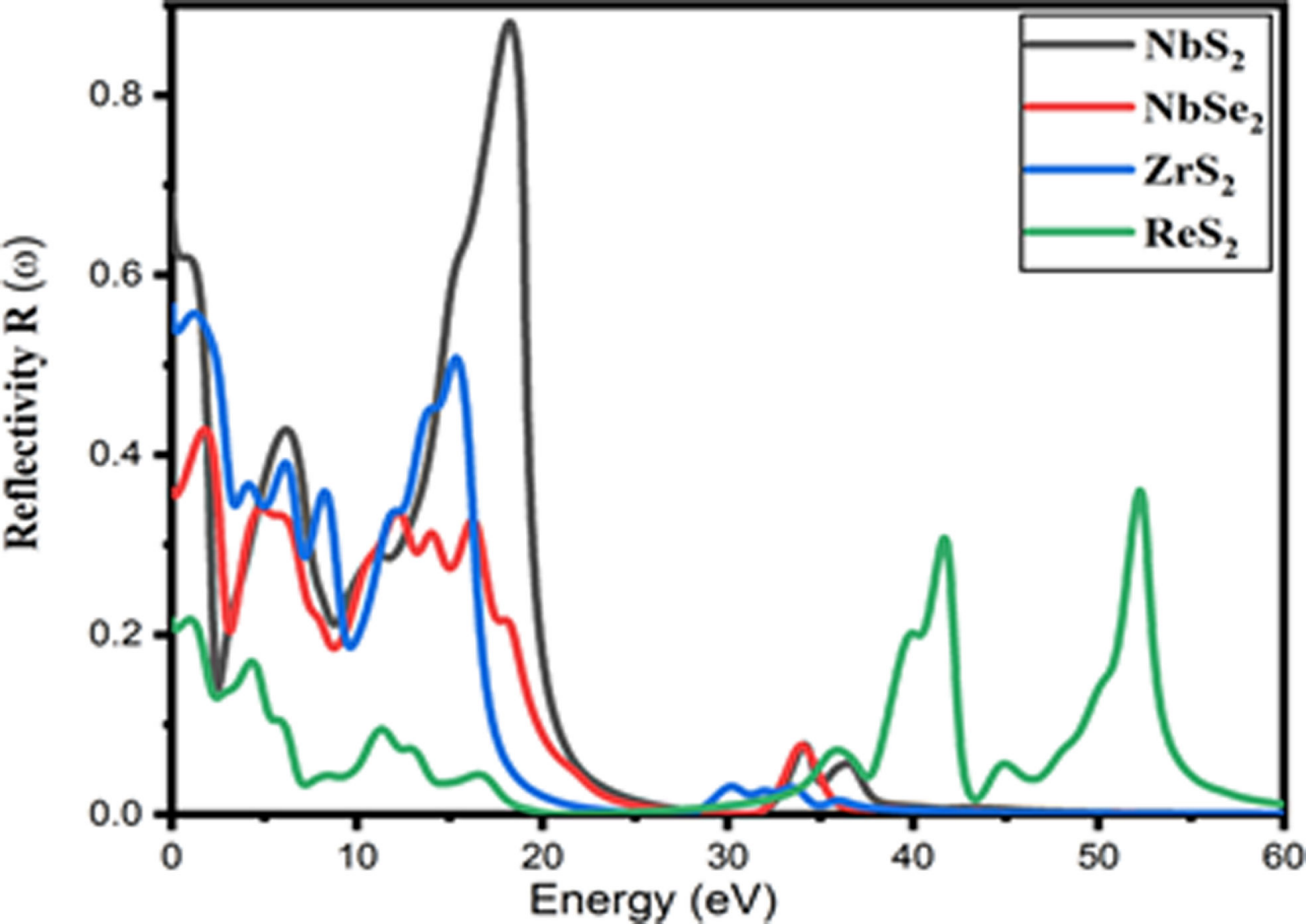
Reflectivity of two-dimensional NbS_2_, NbSe_2_, ZrS_2_ and ReS_2_ TMDC materials.

A semiconductor material’s reflectivity can be ascertained from its surface behaviour. Figure 9 displays the reflectivity response of the two-dimensional NbS_2_, NbSe_2_, ZrS_2_ and ReS_2_ TMDC materials. The reflectivity peaks decreased from 0 to 12 eV, then grew significantly up to the energy range of 15−16 eV as shown in figure 9. Maximum reflectance peaks of 0.88 and 0.51 are observed in two-dimensional NbS_2_ and ZrS_2_ when compared with other NbSe_2_ and ReSe_2_ materials. For ReS_2_, a reflectivity of 0.38 has been attained in the 52 eV range. These results show that there is a slight trend towards higher energy levels within the reflectivity peaks. These two-dimensional NbS_2_, NbSe_2_, ZrS_2_ and ReS_2_ TMDC materials are suitable for photocatalytic and photoanode applications, according to the reflectivity results.

## 4. Conclusion

Two-dimensional materials are among the most scientifically accessible materials in material science at the beginning of the twenty-first century. There has been interest in the monolayer TMDC family because these materials, with their high UV absorption efficiency, promote effective photocatalytic degradation of pollutants, making them suitable for wastewater treatment. This reduces reliance on harmful chemical treatments, offering an environmentally friendly alternative. Based on DFT calculations, it is determined that NbS_2_, ZrS_2_, ReS_2_ and NbSe_2_ have zero energy bandgap (*E*_g_). The additional gamma active states that are generated in NbS_2_, ZrS_2_, ReS_2_ and NbSe_2_ materials aid in the construction of the CB and VB resulting in a zero *E*_g_. The maximal optical conductivity and absorbance of two-dimensional TMDCs NbS_2_, ZrS_2_, NbSe_2_ and ReS_2_ shows 2.4 × 10^5^, 2.5 × 10^5^, 2.8 × 10^5^ and 7 × 10^5^ $\Omega^{-1}\;cm^{-1}$ , respectively. The TMDC family, which includes NbS_2_, ZrS_2_, ReS_2_ and NbSe_2_, is a group of materials with improved surface area for light photon absorption. These materials also reduce the rate of recombination of light photons and increase charge transport, making them suitable for use in photocatalytic and photoanode applications. Future work should explore the potential of two-dimensional TMDCs, particularly NbS_2_, ZrS_2_, ReS_2_ and NbSe_2_, in addressing the growing need for sustainable energy and environmental solutions. This study lays the groundwork for experimental research, including the use of these materials as photoanodes in sensitized solar cell fabrication. Additionally, future research should focus on assessing the long-term stability of these materials under UV exposure and investigating their combination with other photocatalysts to improve efficiency in multi-pollutant degradation and solar-driven water purification.

## Data Availability

The supporting data of this study have been uploaded to Dryad [57].
